# Enhanced Atrous Spatial Pyramid Pooling Feature Fusion for Small Ship Instance Segmentation

**DOI:** 10.3390/jimaging10120299

**Published:** 2024-11-21

**Authors:** Rabi Sharma, Muhammad Saqib, C. T. Lin, Michael Blumenstein

**Affiliations:** 1 School of Computer Science, University of Technology Sydney, Broadway, Sydney 2007, Australia or muhammad.saqib@csiro.au (M.S.); chin-teng.lin@uts.edu.au (C.T.L.); michael.blumenstein@uts.edu.au (M.B.); 2 National Collections & Marine Infrastructure, CSIRO, Sydney 2007, Australia

**Keywords:** instance segmentation, maritime surveillance, convolutional neural network, attention

## Abstract

In the maritime environment, the instance segmentation of small ships is crucial. Small ships are characterized by their limited appearance, smaller size, and ships in distant locations in marine scenes. However, existing instance segmentation algorithms do not detect and segment them, resulting in inaccurate ship segmentation. To address this, we propose a novel solution called enhanced Atrous Spatial Pyramid Pooling (ASPP) feature fusion for small ship instance segmentation. The enhanced ASPP feature fusion module focuses on small objects by refining them and fusing important features. The framework consistently outperforms state-of-the-art models, including Mask R-CNN, Cascade Mask R-CNN, YOLACT, SOLO, and SOLOv2, in three diverse datasets, achieving an average precision (mask AP) score of 75.8% for ShipSG, 69.5% for ShipInsSeg, and 54.5% for the MariBoats datasets.

## 1. Introduction

**Figure 1:**
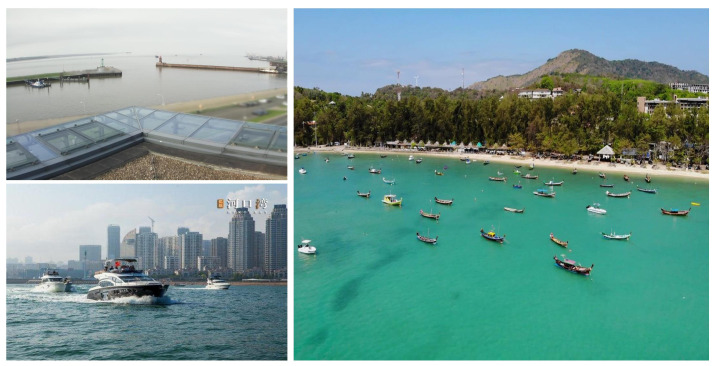
Sample images of small ships in different scenarios shown from three marine datasets such as ShipSG (**Top-left**), MariBoats (**Botton-left**), and ShipInsSeg (**Right**).

Instance segmentation offers a precise and effective means of segmenting objects by labeling pixels with different colors and thus identifying the different instances of the object belonging to the same category. It can locate all individual objects and outline and categorize them [1], unlike object detection, which merely sets a window over them. In relation to marine ship instance segmentation, it classifies individual ships into different categories and obtains accurate ship positions beneficial for marine surveillance and ocean traffic management. Instance segmentation has been extensively studied and applied in various fields, including video surveillance [2,3], maritime surveillance [4,5,6,7], and biomedical applications [3,8]. However, it is observed that, particularly for small objects, instance segmentation tends to underperform compared to other segmentation methods [9]. In the real world, marine ships are captured from a vast distance, which results in fewer pixels per object. Small ships also feature in cluttered backgrounds and indistinct boundaries as shown in Figure 1. Traditional instance segmentation techniques using convolution, pooling, sampling, etc., have difficulty in extracting the important features of small objects. As a result, small ships are missed due to inaccurate detection and segmentation. Thus, the instance segmentation of small objects is a challenging task.

Limited research exists on small ship instance segmentation, with most work focusing on small object detection and semantic segmentation in remote sensing [10,11], infrared images, SAR images and visible images [12]. However, limited methods are proposed to address the small ship instance segmentation of visible images. It can be noted that instance segmentation techniques perform well on medium and large objects but struggle with small objects.

The goal of this research is to improve the detection and segmentation of small ship instances in visible light images and complex backgrounds as shown in Figure 1. Our conjecture is that different layers lack semantic information during the feature extraction process for small ships; thus, we need to improve different semantic information for various levels. Hence, this paper proposes a small ship instance segmentation method with an enhanced Atrous Spatial Pyramid Pooling (ASPP) feature fusion module. The proposed method is important because it can effectively enhance different layers of missing semantic information, which results in the more accurate segmentation of small ship instances compared to single enhancement methods. Specifically, the feature extracted from the ResNet network consists of four feature layers: F1, F2, F3, and F4. These feature layers capture different semantic information. The lowest layer, F1, of the model includes features such as boat contour and texture. It comprises low-level semantic information that may be used for object detection and segmentation. The middle layers, F2 and F3, contain lower-level and high-level semantic information, such as the parts of ships and global patterns. In the high-level semantic information obtained from the F4 layer, objects may be classified based on whether they are ships or buoys. However, these feature layers are unable to detect small ships, due to the complex background. Additionally, when segmenting smaller ships located further away, the traditional FPN generates redundant, meaningless information by fusing the feature maps from high-level and low-level semantic information about the smaller ships, leading to information loss. To solve this, we propose a novel solution for small ship instance segmentation to enhance performance and accurately segment small ships in complex environments. Furthermore, it effectively captures the fine details of small ships, which are often difficult to detect and segment in long-distance visible images.

Our contribution can be summarized as follows:

- Our proposed enhanced ASPP Feature Fusion module uses dilated convolutions with large receptive fields which are able to capture features from a broader area, without increasing the number of parameters. It contains novel attention fusion techniques that effectively capture channel-wise features across channels and spatial relationships based on their coordinates. Finally, we learn the spatial location and suppress irrelevant ones. It can segment small ships to perform instance segmentation tasks that are missed due to weak feature information.
- We conduct extensive experiments using three maritime ship datasets: MariBoats, ShipSG, and ShipInsSeg. Our experimental results demonstrate the superior performance of our enhanced ASPP feature fusion module in accurately segmenting small ships across diverse datasets.

The paper is structured as follows: Section 2 provides an overview of the relevant literature on instance segmentation, attention mechanisms, and maritime surveillance applications. In Section 3, the proposed methodology is discussed. Section 4 presents the experiment results. Section 6 concludes with the study’s findings.

## 2. Related Work

This section briefly overviews the earlier approaches and developments, including instance segmentation, attention mechanisms, and marine surveillance applications.

### 2.1. Instance Segmentation

Instance segmentation techniques are broadly divided into two techniques, i.e., one-stage and two-stage instance segmentation, which are briefly discussed as follows.

#### 2.1.1. One-Stage Instance Segmentation

These are adapted from single-stage object detection techniques [13,14,15]. Previously, instance segmentation is based solely on object location, category, and instance mask. CenterMask [16] predicts the positions of bounding boxes and object centers to estimate the shape and instance location. Using the mask head, precise masks are generated that capture object boundaries and shapes. The fusion of these two sources of information achieves instance segmentation by handling both the image context and object details. Polarmask [17] enhances efficiency by implementing polar coordinates to handle the two concurrent tasks, such as center classification and dense regression. By incorporating the blender module, Blendmask [18] effectively fuses high-level context and low-level details, resulting in improved precision in instance segmentation. SOLO [19] employs a technique that does not rely on bounding box supervision. By utilizing a fully convolutional feature map, it processes images and provides outputs connected to their categories and instance masks. One-stage instance segmentation is less complex and quicker than the two-stage method but has lower accuracy.

#### 2.1.2. Two-Stage Instance Segmentation

The core idea behind two-stage instance segmentation follows a technique called “detect then segment”, where the object is first detected and then segmented to generate an instance mask. Mask R-CNN builds upon Faster R-CNN [20] by adding an FCN network and ROI Align for instance segmentation, a baseline approach for instance segmentation. To improve the feature extraction backbone using the adaptive fusion ROI regional feature, the PANet [21] method introduces a bottom–up route expansion based on Mask R-CNN. Mask Scoring R-CNN [22] is a modified version of Mask R-CNN that adds a mask-IoU branch to improve the quality of the mask and increase the confidence score. To improve the computational cost and accuracy, cascade techniques are used in the detection and instance segmentation task called Cascade R-CNN (Cascade Mask R-CNN) [23], which contains three branches of classification, and the detection head has an intermediate connection and the last branch has an added segmentation task. Tensormask [24] employs a sliding window technique to partition objects into smaller sections, ensuring a fixed number and portion of sections per pixel. Some other methods [25,26,27] create object masks by categorizing pixels into any number of instances that are present in the image. The two-stage techniques are superior in terms of accuracy and well-developed algorithms. Due to its advantage, we use Cascade Mask R-CNN as the benchmark for the small ship instance segmentation task.

### 2.2. Attention Mechanisms

In recent years, attention mechanisms have been intensively explored and implemented to address different computer vision tasks, such as classification [28,29,30,31], object detection [28,29,30,31], semantic segmentation [31], and instance segmentation [30], due to its simplicity, robustness, and easy plug-and-play nature. Improving feature extraction and the performance of neural networks is the primary objective for different attention mechanisms (channel-wise, spatial, dimensional-wise, etc.). In Squeeze and Excitation (SE) [28], each two-dimensional feature map is squeezed to effectively build channel dependency, focusing on channel information instead of location information. Efficient Channel Attention (ECA) [29] was proposed to improve the excitation module to minimize the model’s complexity. To address the limitations of spatial information in SE, the Convolutional Block Attention Module (CBAM) [30] was proposed to focus on both channel and spatial regions. However, CBAM mechanisms compute independently and ignore domain relationships. Coordinate attention (CA) [31] addresses the limitation of SE, which avoids positional information, and CBAM, which disregards long-term dependencies. Inspired by the following attention mechanisms, we propose a novel fusion technique to increase generalization, focus on various aspects of input data, and acquire robust features to handle the challenges of marine scenarios.

### 2.3. Marine Surveillance Applications

Marine surveillance is an active research area in computer vision. In recent times, efforts have increased significantly to safeguard our coastline from activities such as smuggling, unauthorized immigration, and assaults on maritime vessels. As far as surveillance [32,33] is concerned, traditional remote sensing methods can be useful, but ships must rely on specialized observatories and communication equipment. This costly equipment can fail or be purposely turned off. These systems heavily rely on human supervision. Almost 80% of this human perception data comes from human vision. However, human vision is inherently prone to error, as humans may misinterpret or misjudge visual information. Vision technology can make surveillance safer and more cost effective by helping intelligent systems provide better visual information. By utilizing vision-based technologies for ocean monitoring, a nation can secure its interests and safeguard the privacy of essential defense information.

In the marine environment, object detection is valuable for a range of safety purposes, including the identification of sharks [34], population estimation [35], and protection against drone threats [36]. Prasad et al. [37] used straightforward video processing techniques to handle problems like horizon recognition, static background removal, etc. The several processing stages used in this approach make it slow. When MobileNetV2 and SSD are used for detection tasks, the ship identification and classification are enhanced [38] with a mean average precision of 92% with a speed of 15 frames per second. Similarly, a study conducted by Li et al. [39] identified and classified ships by incorporating remote sensing images. Until now, object detection using deep learning technology has achieved high accuracy and speed.

Few studies have been conducted using instance techniques for marine surveillance in recent years. To better comprehend the marine environment, instance masks are constructed with the aid of global mask heads [4]. This method makes use of both global and semantic information. Zequn et al. [40] proposed a global and local attention module that enhances segmentation performance by preserving global and semantic information. Different from the aforementioned works, we concentrate on small ships in visible images and enhance the instance segmentation network for better segmentation results.

### 2.4. Small Object Segmentation

Research on small object instance segmentation is minimal; most of the work was focused on object detection and the segmentation of large objects. Using data augmentation, Yang et al. [41] created a small object dataset and developed a real-time semantic segmentation model for small objects in urban scenarios. Ref. [42] proposes a multi-task system with a UNet backbone for preserving resolution in infrared tiny object recognition and segmentation. In [43], a perceptual generative adversarial module is introduced to handle tiny object detection by extending their representation to super-resolution. For remote sensing applications, the semantic segmentation and segmentation of small objects in aerial images is very important. Li et al. [11] introduced a class-incremental learning network and a diversity distillation loss to address semantic segmentation for small objects. Ma et al. [10] introduced foreground activation-driven tiny object semantic segmentation to enhance weaker features for small objects. Chen et al. [44] improved the instance segmentation of small objects by incorporating an auxiliary segmentation model alongside a box attention module. In [12], a new small ShipInsSeg dataset was proposed to solve the problem of segmenting small ships using a dual activation branch and dual mask branch. However, there has been limited study on small ship instance segmentation in visible images.

## 3. Proposed Method

The overall structural diagram of our proposed model is discussed in this section. We then discuss our enhanced ASPP feature fusion module along with other attention mechanisms that were used. We conclude with the loss function used for training.

### 3.1. Overall Architecture

**Figure 2:**
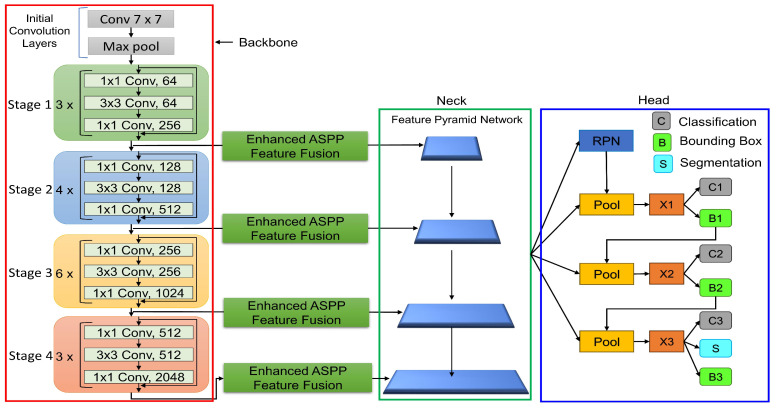
The overall architecture of the proposed framework. The architecture contains three parts: the backbone (a red rectangle box), the neck (a green rectangle box), and the head (a purple rectangle box). The backbone contains the ResNet architecture for feature extraction at different stages. The neck contains a Feature Pyramid Network (FPN) that captures objects from different scales. The head outputs three tasks, namely, classification, bounding box, and segmentation (instance mask). Our proposed module, enhanced ASPP Feature Fusion, is shown in the green block.

Figure 2 depicts the architecture of our proposed technique, which is based on the Cascade Mask R-CNN [23] framework. It is made up of three main components: the backbone, the Feature Pyramid Network (FPN) [45], often known as the neck, and the detection and segmentation head. In Figure 2, the red rectangle box shows the backbone network. The input X ∈$R^{3HW}$ is fed to the backbone network for feature extraction. Initially, we used 7 × 7 convolution layers with a stride of two, which provides a larger receptive field to capture basic features like edges and patterns, and the max pooling layer was used for dimension reduction and to reduce the computational cost. Next, the model contains four stages, from one to four. Each stage consists of a different number of bottlenecks as shown in Figure 2. The bottleneck contains a set of three convolution layers: 1 × 1 is used to reduce the feature dimension to make the network efficient, 3 × 3 is used for feature extraction to capture complex patterns, and lastly, 1 × 1 is used to expand the feature to higher dimensions to make it forward to the next stage. Additionally, each bottleneck adds skip connections to avoid the vanishing gradient problem. Stage 1 (green block) consists of 3 bottlenecks, which capture edges, corners and textures. Stage 2 has 4 bottlenecks, which can extract more complex features. Due to the increase in network depth at Stage 3, which has 6 bottlenecks, more higher-level features can be extracted. At this stage, the feature maps encode semantic information better than spatial information, making them useful for detection and classification. The final Stage 4 includes 3 bottlenecks with the most abstract features and helpful information. The output features of each stage, such as F_i_∈ {256, 512, 1024, 2048}, are compressed and refined by our enhanced ASPP feature fusion module. The enhanced features are sent to the Feature Pyramid Network.

The top–down pathway of the FPN (green rectangle box), where the topmost layer denotes the feature maps, has the lowest spatial resolution but the highest semantic information. These layers are upsampled (by a factor of 2) to match the size of the feature map from the preceding stage. A lateral connection merges with these upsampled feature maps using a 1 × 1 convolution layer to ensure that the channel dimensions are the same. Additionally, the primary task of FPN is to refine features and detect ship instances at different scales. Finally, the detection and segmentation head shown in the blue rectangle box is also called the cascade detection and segmentation head. The FPN sends its output to the cascade detection and segmentation head, where the Regional Proposal Network (RPN) first locates anchors for ship instances box_0_ in Equation (1):

(1) $$\begin{array}{cc} box_{0} & =feature_{\mathrm{RPN}}\left(P\right) \end{array}$$

where RPN is computed by feature_RPN_(*P*).

Following anchor candidates box_0_, we calculate bounding boxes using the Cascade R-CNN structure. Note that Cascade R-CNN employs bounding boxes in one stage as anchors for the ROIAlign module in the following stage. Such a design consists of three network heads, namely {x1, x2, x3}, trained with increasing IoU thresholds of 0.5, 0.6, and 0.7, respectively. The RPN network generates the proposals and then passes them to the ROI pooling layer. The three network heads {x1, x2, x3} receive the output of the ROI pooling layer as input and then perform two prediction tasks, namely, classification (C1, C2, and C3) and bounding boxes (B1, B2, and B3). In addition to the third network head, we also compute instance segmentation task (S).

### 3.2. Enhanced ASPP Feature Fusion

**Figure 3:**
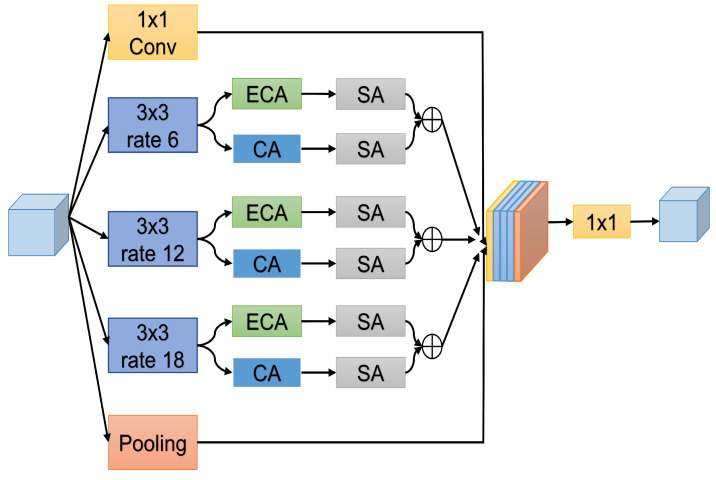
Structural diagram of proposed enhanced ASPP feature fusion, where we use three convolution layers with kernel size 3 × 3 having dilation rates of 6, 12, and 18. The output is fed to two parallel attentions, Efficient Channel Attention (ECA) and coordinate attention (CA), followed by spatial attention (SA), for feature enhancement. It focuses on important feature maps and concatenates them. Furthermore, a 1 × 1 convolution layer and an average pooling layer are used. Finally, all outputs are concatenated and pass through a 1 × 1 convolution layer.

We revisit the proposed Atrous Spatial Pyramid Pooling (ASPP) [46], which applies four parallel atrous convolutions on top of the feature map at varying dilation rates to solve the problem of semantic segmentation. The success of spatial pyramid pooling [47,48] encouraged ASPP, which demonstrated that resampling features at various scales may enhance the accuracy and efficient categorization of areas of any size. We propose an enhanced ASPP feature fusion module for segmenting small ships and improving performance as illustrated in Figure 3. Our module incorporates a 1 × 1 convolution layer to preserve the original dimensions. We use three dilated convolutional layers with a kernel size of 3 × 3 and different dilation rates of 6, 12, and 18 to capture information from different resolutions effectively. The advantage of using different dilation rates of 6, 12, and 18 is that they can aggregate information from different distances across the input feature maps, which helps to improve the performance of ship instance segmentation as well as segment smaller ships. Additionally, increasing dilation rates improves performance without increasing the number of parameters. Both efficient channel-wise attention and coordinate attention use the output feature maps from three dilation convolution layers to focus on important channels and suppress less useful ones, as well as to capture long-range dependencies and emphasize positional information. By applying spatial attention, we further focus on the spatial location of small ships, which is essential for segmenting small ship instances more clearly. Combining these attention mechanisms makes our model better able to handle complex marine scenarios. Finally, the resulting feature maps of all branches are concatenated and passed via a 1 × 1 convolution layer to make the input and output dimensions the same.

### 3.3. Efficient Channel Attention

**Figure 4:**
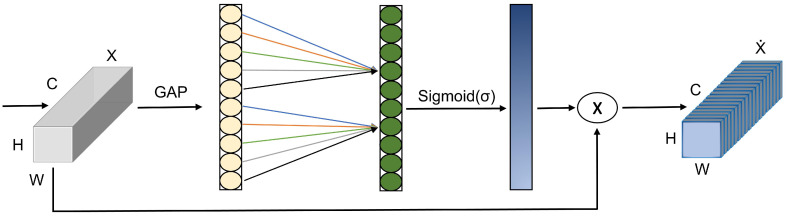
Structural diagram of the Efficient Channel Attention (ECA) mechanism, where C refers to channels, H refers to height, and W refers to width. ⨂ represents element-wise multiplication.

The attention mechanism in deep learning models replicates human visual or cognitive concentration by focusing only on specific input data. The distribution of weights depends on the level of attention received by each part rather than being shared equally. As a result, the model focuses on important information for the segmentation task, making it more accurate at segmenting things. Popular attention mechanisms used in various applications are SE and CBAM. The ECA [29], as shown in Figure 4, is a powerful channel attention technique developed to capture cross-channel dependencies and improve convolutional neural network feature representation.

To gather overall context information for each channel in an input image, X[C, H, W], the ECA utilizes global average pooling (GAP), where “C” represents the number of channels, and “H” and “W” represent the dimensions (height and width) of the feature map. The expression of GAP is defined in Equation (2):

(2) $$\begin{array}{c} GAP\left(Y\right)=\frac{1}{HW}\sum_{x=1}^{Height}\sum_{y=1}^{Width}Y\left[c,x,y\right]\;\forall c\in \left\{1,.,B\right\} \end{array}$$

In the above equation, Y[c,x,y] represents the (x,y)th channel c element in the input feature map Y. The output of GAP is a vector of the channel B dimension. Using a 1D convolution layer, the ECA attention module captures local channel relationships. The ECA attention mechanism dynamically determines the size of the convolution kernel for the 1D convolution layer.

The term "Fconv1D” describes a convolution operation with a k-size kernel.

ECA can handle a different number of channels and better understand how channels relate to each other in a specific location. The size of the convolution kernel, represented by k, is calculated using Equation (3):

(3) $$Kernel\left(k\right)={|\;\frac{log_{2}\left(C\right)+b}{\gamma }|}_{\mathrm{odd}}$$

where k denotes the kernel size, and C represents the channel dimension. γ and b are set to 2 and 1, respectively. Including the ECA attention mechanism in convolutional neural networks enhances feature representation by capturing connections between different channels. High computing efficiency, a small number of parameters, and smooth interaction with current convolutional neural networks are some of its advantages.

### 3.4. Coordinate Attention

**Figure 5:**
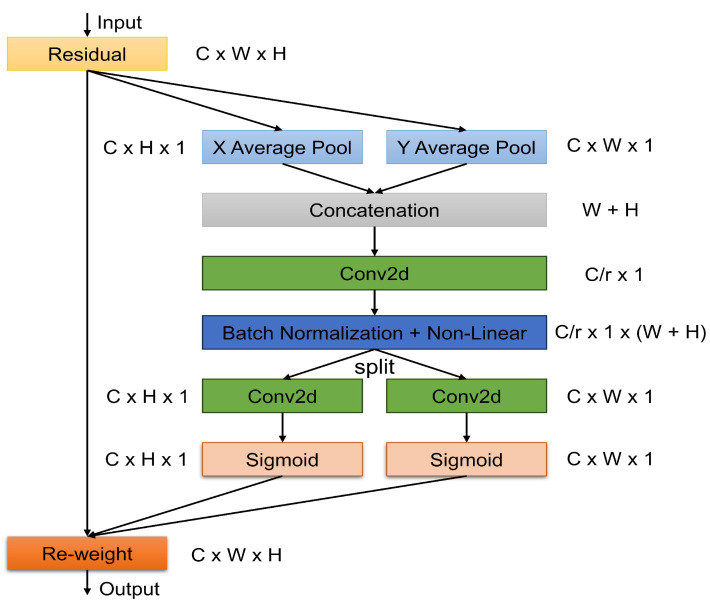
Structural diagram of coordinate attention (CA).

The Squeeze and Excitation (SE) block uses global pooling to obtain global spatial information and simulate cross-channel relationships while ignoring the significance of positional information. CBAM uses convolutions to capture local relationships but lacks the capability to model long-range dependencies. To address these problems, coordinate attention (CA), a new attention mechanism, is proposed as shown in Figure 5, which incorporates positional information into channel attention. This enables the network to concentrate on large, significant regions at a minimal computational cost. The coordinate attention mechanism encrypts coordinate information and channel correlation in two phases: coordinate information embedding and attention generation. First, to incorporate the coordinate information, global pooling on the inputs X along the horizontal and vertical directions generates two different attention maps, Feature_h_ and Feature_v_, which represent both the horizontal and vertical coordinate attention as defined in Equations (4) and (5):

(4) $$\begin{array}{cc} Feature_{h}= & Pool_{h}\left(X\right) \end{array}$$

(5) $$\begin{array}{cc} Feature_{v}= & Pool_{v}\left(X\right) \end{array}$$

where the global pooling operations are horizontally and vertically denoted as $Pool_{h}$ and $Pool_{v}$, respectively. Second, to create CA, we have to aggregate the two attention maps $x_{h}$ and $x_{v}$, and apply point-wise convolution and non-linear activation. Equations (6) and (7) are given in the following:

(6) $$\begin{array}{cc} x_{h}= & \delta [Point_{1}\left(Feature_{h}\right)] \end{array}$$

(7) $$\begin{array}{cc} x_{v}= & \delta [Point_{2}\left(Feature_{v}\right)] \end{array}$$

where δ is the non-linear activation. $Point_{1}$ and $Point_{2}$ refer to the point-wise convolution operation. Additionally, using the point-wise convolution operation helps with channel scaling and applying the sigmoid function for $x_{h}$ and $x_{v}$, which creates accurate coordinate information. $Weight_{\mathrm{ca}}$ is utilized as the attention weights after expanding and multiplying $x_{h}$ and $x_{v}$. The expression can be calculated using Equation (8):

(8) $$\begin{array}{c} Weight_{\mathrm{ca}}=X\cdot \sigma \left[Point_{3}\left(x_{h}\right)\right]\cdot \sigma \left[Point_{4}\left(x_{v}\right)\right] \end{array}$$

where σ refers to the sigmoid function. $Point_{3}\left(x_{h}\right)$ and $Point_{4}\left(x_{v}\right)$ denote point-wise convolution operations. Lastly, $Weight_{\mathrm{ca}}$ is the final attention weight for CA.

### 3.5. Spatial Attention

**Figure 6:**
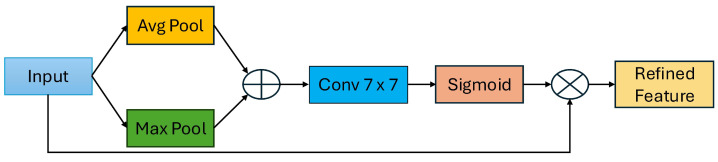
Structural diagram of spatial attention (SA).

After applying channel-wise and coordinate-based attention, spatial attention enhances the model’s focus on the important features reside spatially. As shown in Figure 6, consider the input feature I^CxHxW^. We apply max pooling and average pooling across the channel axis to create 2D feature maps with dimension $R^{1xHxW}$. After concatenating the two feature maps using ⨁, it generates effective feature maps with dimension $R^{2xHxW}$. To create a spatial attention map, a convolution layer with a kernel size of 7 × 7 is used, and the sigmoid function is applied. Finally, Equation (9) is given as follows:

(9) $$\begin{array}{c} SA=\sigma (Conv(concat(Maxpool(InFeat),\\ Avgpool(InFeat))) \end{array}$$

### 3.6. Loss Function

For the training loss function of our network, the cascade structure to compute the loss function contains four parts: regional proposal network (RPN), classification, regression, and mask. The three network heads are calculated with different IoU values ranging from {0.5, 0.6, 0.7}. In the first and second heads, the loss function is computed for classification and regression tasks. In the third head, we calculate the loss function of three tasks, namely, classification, regression, and mask. The network heads are interconnected via B1, B2, and B3 as shown in Figure 2. Finally, the entire model loss function is defined in Equation (10):

(10) $$Loss_{all}=Loss_{RPN}+Loss_{cls,x}+Loss_{box,x}+Loss_{mask,3}$$

where x = 1, 2, 3 for the three network heads. The classification and regression loss functions are denoted by $Loss_{cls}$ and $Loss_{box}$. $Loss_{mask}$ represents mask loss.

## 4. Experiment Analysis

In this section, we evaluate the performance of our proposed enhanced ASPP feature fusion module for small ship instance segmentation. First, we discuss the datasets used for our experiments and the evaluation metrics used in our studies. The hyperparameters used during training are then discussed. We examine the model’s performance through ablation studies conducted on three datasets.

### 4.1. Dataset and Metrics

We evaluate the performance of our proposed method on three marine ship datasets, namely, MariBoats [41], ShipSG [49], and ShipInsSeg [50]. First, we discuss the marine datasets used for our experiments. Table 1 provides comprehensive details on the small ships in a concise way. Second, we cover the implementation details used for training. The marine ship datasets are briefly explained in the following:

#### 4.1.1. ShipSG Dataset

The ShipSG dataset, generated by two cameras at Fischereihafen-Doppelschleuse lock in Bremerhaven, Germany, contains 3505 images of 11,625 ships in polygon format, annotated with seven classes like cargo, law enforcement, passenger/pleasure, special1, special2, tanker, and tug. Ship masks were manually annotated using LabelMe [51]. The dataset has a resolution of 1333 × 800 pixels, with 80% used for training and 20% for validation, making it suitable for instance segmentation tasks.

#### 4.1.2. MariBoats Dataset

In this study, we use the MariBoats dataset consisting of 6271 images that contain only one class, i.e., boat, with 15,777 boat annotations. The dataset has images that were taken from 13,717 boat images found on the Google search engine using certain keywords like cargo ships, fishing boats, and more. Images with duplicate information, poor quality, fuzzy, or unrelated to boats are removed. The dataset is freely accessible and suitable for instance segmentation.

#### 4.1.3. ShipInsSeg Dataset

Our ShipInsSeg dataset contains 5116 images with only one class, i.e., boat. The image has a 1280 × 720 resolution, and the dataset is split into 80% for training and 20% for testing. These images are collected from YouTube videos and made available under the Creative Commons license. We use the LabelMe annotation tool to annotate the polygon format for our instance segmentation task. The dataset is complicated and features various weather conditions, and occlusions such as sun rays, water reflection, crowded ships, and big waves.

### 4.2. Experiment Setup

The study uses MMDetection on Ubuntu 18.04.6 LTS, PyTorch, CUDA, and CUDNN to train models on two GPUs, NVIDIA Quadro P6000. Randomly selected training samples are used for marine datasets. To improve efficiency, the backbone network’s weight is pre-trained and stochastic gradient descent (SGD) is used as an optimization strategy. The models are trained for 12 epochs, with a weight decay of 0.0001 and a momentum of 0.9. After the 8th and 11th epochs, the learning rate is dropped by 0.1. All other MM detection-related hyperparameters remain unchanged.

### 4.3. Experiment Results

To assess the effectiveness of ship instance segmentation models, we employ MS COCO as a quantitative metric system, namely AP_50_, AP_75_, AP_S_, AP_M_ and AP_L_ to evaluate the marine ship instance segmentation. Average precision (AP) is a commonly used measurement for evaluating instance segmentation. Intersection over Union (IoU) plays an important factor in the evaluation as shown in Equation (11):

(11) $$IoU=\frac{intersect(X_{t},Y_{t})}{union(X_{t},Y_{t})}$$

In Equation (11), X_t_ and Y_t_ represent target prediction and ground truth. The area of intersection of the target prediction and ground truth is intersect(X_t_,Y_t_) and union(X_t_,Y_t_) represents the union area for both prediction and ground truth. We calculate AP_50_ and AP_75_ ranges from the thresholds of 0.5 and 0.75, respectively.

AP_S_, AP_M_, and AP_L_ are used to show how accurately objects of different sizes are measured, i.e, AP_S_ represents small objects ($\mathrm{area}<32^{2}$), AP_M_ represents medium-sized objects ($32^{2}<\mathrm{area}<96^{2}$), and AP_L_ represents large objects ($\mathrm{area}>96^{2}$), respectively. Additionally, we report the evaluation metrics for both mask AP and box AP, respectively.

Precision is measured by dividing true positives (properly anticipated positive cases) by the total of true positives and false positives in Equation (12):

(12) $$\mathrm{Precision}=\frac{\mathrm{TP}}{\mathrm{TP}+\mathrm{FP}}$$

To calculate recall, we divide TP by the sum of TP and FP (positive examples ignored by the model) as shown in Equation (13):

(13) $$\mathrm{Recall}=\frac{\mathrm{TP}}{\mathrm{TP}+\mathrm{FN}}$$

**Figure 7:**
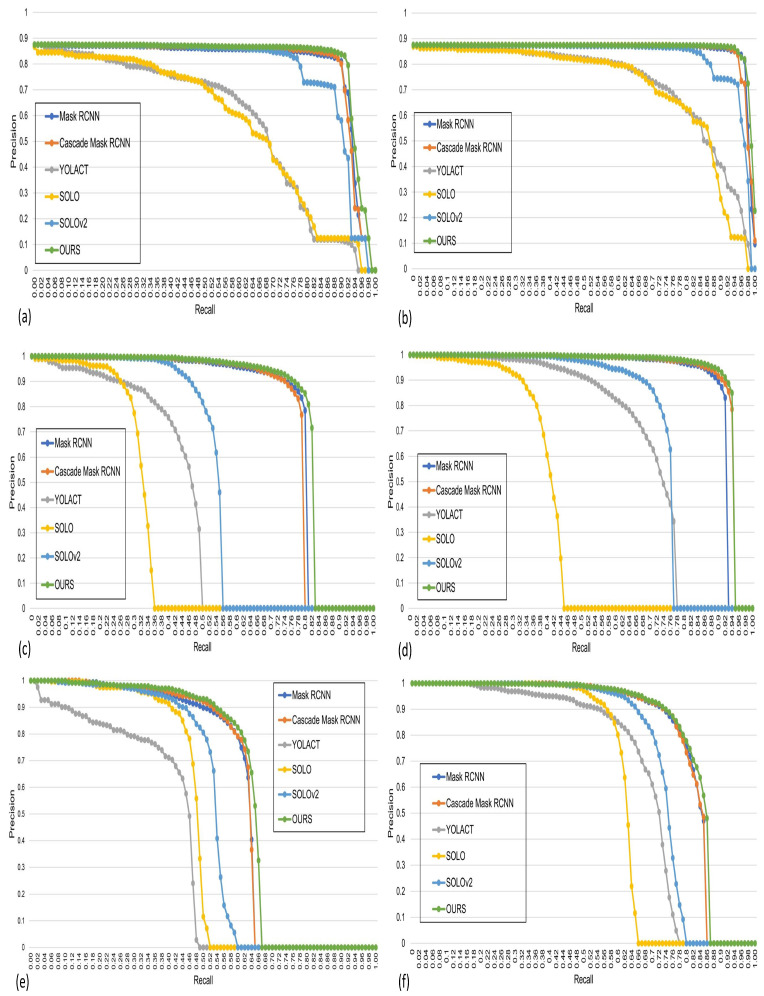
PR curves are displayed for all classes according to C75 and C50. The 1st row shows the PR curves based on C75 (**a**) and C50 (**b**) for the ShipSG. The curves for the ShipInsSeg dataset are shown in the 2nd row according to C75 (**c**) and C50 (**d**), and the MariBoats curves are shown in the 3rd row based on C75 (**e**) and C50 (**f**). Our proposed model PR curves are displayed in green (best viewed in color).

**Figure 8:**
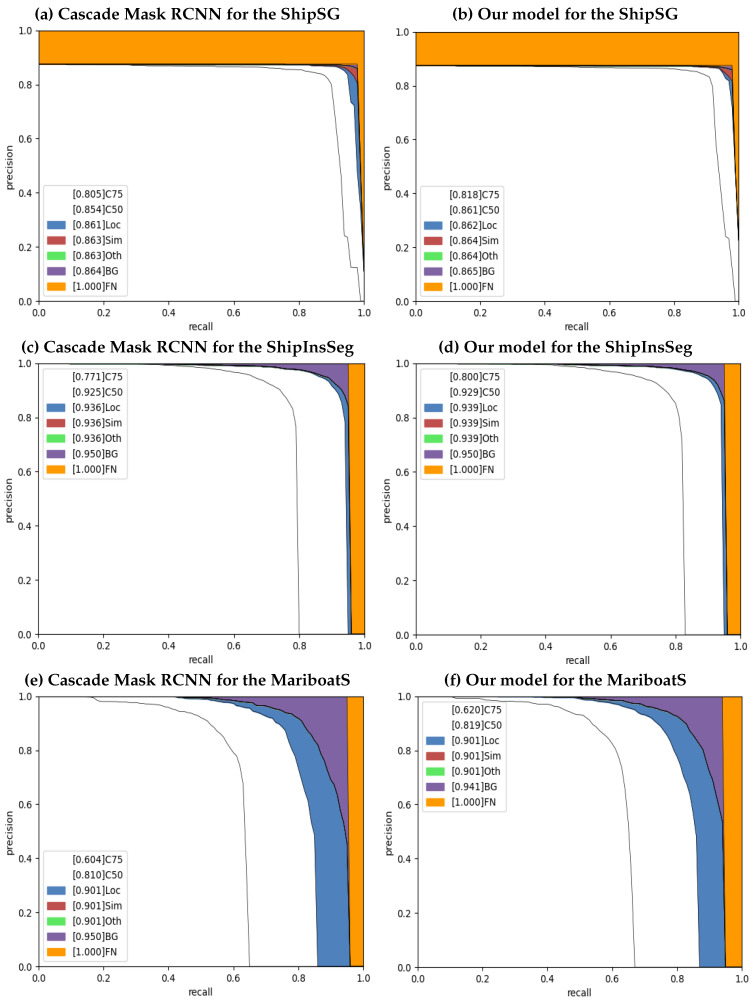
Error analysis via PR curves for three marine datasets. The 1st row shows the error analysis for ShipSG: (**a**) Cascade Mask RCNN, and (**b**) ours. The 2nd row displays the error analysis PR curve for ShipInsSeg (**c**) representing Cascade Mask RCNN and (**d**) ours. Finally, the 3rd row shows the MariBoats error analysis PR curve: (**e**) Cascade Mask RCNN and (**f**) ours.

Figure 7 and Figure 8 illustrate the PR curves and error analysis curves of different state-of-the-art models for the three marine datasets. Furthermore, we generate precision–recall (PR) curves for all classes using the C75 and C50 metrics at IoU thresholds of 0.75 and 0.50, respectively.

### 4.4. Performance Evaluation of Instance Segmentation

**Results on ShipSG dataset:**

Table 2 shows the results of comparing our approach with other models on the ShipSG dataset. We compare our method with five instance segmentation methods (one stage and two stage), namely, Mask RCNN [1], Cascade Mask RCNN [24], YOLACT [52], SOLO [19], and SOLOv2 [53]. Additionally, we also compare our method using four attention mechanisms, namely, CBAM [30], ECA [29], SE [28], and CA [31]. Our plug-and-play module is deployed in alternative two-stage instance segmentation methods, such as Mask RCNN, to test the accuracy using the ResNet-50 and ResNet-101 backbones as shown in Table 2, and its generalization capabilities are calculated.

Table 2 shows the comparison results. We note that our method considerably enhances the segmentation performance of the Cascade Mask RCNN (marked in bold). Specifically, when compared to the baseline model, Cascade Mask RCNN with ResNet101 as the backbone, our method outperforms it by 1.5% for mask AP. We observe that when we plug in our proposed module in Mask RCNN using the ResNet50 and ResNet101 backbone networks, there is a significant improvement of 1.1% and 1.4% for mask AP. Furthermore, our method surpasses the other state-of-the-art models. For the segmentation of small ships (AP_S_), our approach improves performance from 56.50% to 62.20%. This indicates our method’s superior feature extraction capabilities for small ships, outperforming the other models. Our approach significantly increases the segmentation performance of small ships. Table 2 also shows that our method reduces missed ship detection and segmentation while increasing the recall rate AR of different object scales.

Figure 7 evaluates the PR curve for five benchmark instance segmentation models, Mask RCNN, Cascade Mask RCNN, YOLACT, SOLO, SOLOv2, and ours, for the ShipSG dataset, where the first row represents C75(a) and C50(b), respectively. The graph shows that the proposed model considerably outperforms the state-of-the-art models, represented by the green color, followed by the Mask RCNN and Cascade Mask RCNN models. Since all the models have high accuracy, successful predictions have minimal false positives. Furthermore, we observe that the recall values lie roughly between 0.95 and 0.99 for C75, and 0.98 and 1.00 for C50, which shows that our model performs best for both metrics compared with the other models. Our model maintains high precision with high recall, as seen in Figure 7, proving its robustness and stability in ship instance segmentation.

**Results on ShipInsSeg dataset:**

Similar to ShipSG dataset, we compare five instance segmentation algorithms for the ShipInsSeg dataset, which mostly contains images of small- and medium-sized ships. Furthermore, our approach is evaluated by comparing four attention mechanisms, namely, CBAM, ECA, SE, and CA to comprehend the effectiveness of various attention mechanisms. Table 3 shows that our method significantly outperforms the other models, attaining a performance gain of 1.3% compared to the second-ranked method (Cascade Mask RCNN with backbone as ResNet-101). Moreover, we note that our method performs significantly better on other COCO metrics like AP_50_, AP_75_, AP_S_, AP_M_, and AP_L_. For small ships, the AP_S_ improves from 47.8% to 50.5%, resulting in better performance for smaller ships. Additionally, the average recall is improved for different object scales as shown in Table 3.

As shown in Table 3, we embed our proposed plug-and-play module into other two-stage instance segmentation models, such as Mask RCNN using the ResNet50 and ResNet101 backbone network, showing that embedding our module achieves a performance gain of 0.1% for both when compared with the baseline models.

Similar to the ShipSG dataset, we demonstrate the PR curve for the ShipInsSeg shown in Figure 7 in the second row for C75(c) and C50(d). It can be seen that our model performs well compared with the other models. The recall values for C75 and C50 range between 0.49 and 0.67, and 0.66 and 0.87, respectively, indicating false negatives. The occurrence of false negatives may be related to the model that predicts the background as an object. Therefore, our model has successfully reduced the occurrence of false negatives compared to previous models.

**Results on MariBoats dataset:**

Table 4 demonstrates that our method outperforms existing state-of-the-art models, with a mask AP of 54.5%. This technique beats the second-best method, Cascade Mask RCNN, by 1.0%.

Furthermore, it achieves noticeable performance gains for smaller objects from 9.9% to 10.4% AP_S_ over Cascade Mask RCNN using ResNet-101. When a comparison is made based on COCO metrics, we observe that our method improves the performance for all evaluations like AP_50_, AP_75_, AP_S_, AP_M_, and AP_L_. The performance of AR improves for different object scales as shown in Table 4.

The precision–recall (PR) curve for the MariBoats dataset is shown in Figure 7 in the third row (e) for C75 and (f) for C50. The recall values for C75 and C50 roughly lie between the range of 0.49 to 0.67 and 0.66 to 0.87, i.e., lower than ShipInsSeg, indicating that models have difficulties with false negatives. The potential occurrence of false negatives can be attributed to the model’s prediction of an object as the background.

### 4.5. Error Analysis

To assess our model’s performance, we run an error analysis on these datasets using the COCO error metrics. Figure 8 depicts a comparison of error analysis between the baseline Cascade Mask RCNN and our proposed model for three datasets: ShipSG, ShipInsSeg, and MariBoats. To achieve optimal results, a model’s accuracy and recall should be equal to one. However, in reality, the proposed model and Cascade Mask RCNN suffer from various errors. The metrics used to evaluate the performance of the instance segmentation model are localization errors (Locs), super-category false positives (Sims), category confusion (Oth), total false positives (BGs), and total false negatives (FNs). Figure 8 highlights the model’s limitations, including false negatives, false positives, and localization errors. Table 5 summarizes the error analysis illustrated in Figure 8.

**Error analysis for the ShipSG dataset:**

We compute the error analysis using the PR curve in Figure 8, where (a) and (b) represent Cascade Mask RCNN and our proposed model, respectively. Our model reduces the segmentation error, summarized in Table 5, compared to the baseline model, Cascade Mask RCNN. We find that location error and false negatives are decreased from 0.7% to 0.1% and from 13.6% to 13.5%, respectively, when all error measures are taken into account. Our model achieves an 86.1% correct prediction rate, which is better than Cascade Mask RCNN.

**Error analysis for the ShipInsSeg dataset:**

The error analysis PR curve for ShipInSeg is shown in Figure 8 in the second row, where we compare it with Cascade Mask RCNN (c) as a baseline model and our proposed model (d), respectively. The Table 5 summary makes it evident that our model decreases the segmentation error. Compared with the Cascade Mask RCNN, the correct prediction rises to 92.9%, while the Loc and BG errors drop from 1.4% to 1.1%, and 1.1% to 1%, respectively.

**Error analysis for the MariBoats dataset:**

In Figure 8, the error analysis PR curve for MariBoats is compared to Cascade Mask RCNN (e) and our proposed model (f) in the third row. We note that the correct prediction rate is increased to 81.9% with location error and the BG error is reduced to 8.2% and 4%. However, the FN error increased by 0.9% might be due to the cluttered background.

### 4.6. Object Detection Performance

The relationship between different tasks in instance segmentation, namely, classification, detection, and segmentation, plays a key role in contributing to the model’s learning process. For example, instance segmentation improves object detection performance by providing exact object location information. Similarly, instance segmentation is more accurate because the classification task gives valuable details about object categories. Therefore, we investigated how our method impacted the performance of the object detection task. We analyzed the comparative results shown in Table 6, Table 7, and Table 8 for three marine datasets: ShipSG, ShipInsSeg, and MariBoats, respectively. The dash in Table 6, Table 7, and Table 8 indicates that SOLO and SOLOv2 do not measure box AP since they are one-stage models. It is evident that our method outperformed other models on the object detection task across the three marine datasets on box AP. These results demonstrate that our method is effective not only in instance segmentation but also in object detection tasks. Collaborative learning across interrelated tasks enhances performance and accuracy, addressing complex marine scenarios.

### 4.7. Ablation Study

To evaluate the impact of the enhanced ASPP feature fusion module on instance segmentation, we carried out ablation studies across three datasets, exploring performance across five distinct scenarios. Initially, we evaluated the model without the enhanced ASPP feature fusion module to establish a baseline. Subsequently, we introduced the ASPP feature fusion at various stages of the architecture: after stage 1, after stages 1 and 2, and following stages 1 through 3. In the final scenario, we integrated the module consistently across all stages, from 1 to 4, to observe its full potential. Table 9 illustrates the ablation study for the ShipSG dataset, showing that there was a significant gain of 1.5% on mask AP when we added our proposed module at all stages, compared to without adding our module. Particularly for small ships, we noted that there is a substantial improvement of 5.7% on AP_S_ compared to the baseline model (i.e., without using our module) alongside enhancements in the other evaluation metrics. For the best results, we added this proposed module at all stages. Similarly, for the ShipInsSeg dataset shown in Table 10, we observed that no performance improvements occurred when our module was absent. However, the segmentation performance was improved by including our module at each stage. Integrating our module across all stages achieved a significant improvement of 1.3% on mask AP along with the other COCO evaluation metrics. Although focusing on small ships, there was an increase of 2.7% on AP_S_ compared with no added module. In Table 11, we show the effectiveness of the proposed module for the MariBoats dataset, where there is a performance gain of 1% on mask AP compared with that with no added module.

### 4.8. Qualitative Results

**Figure 9:**
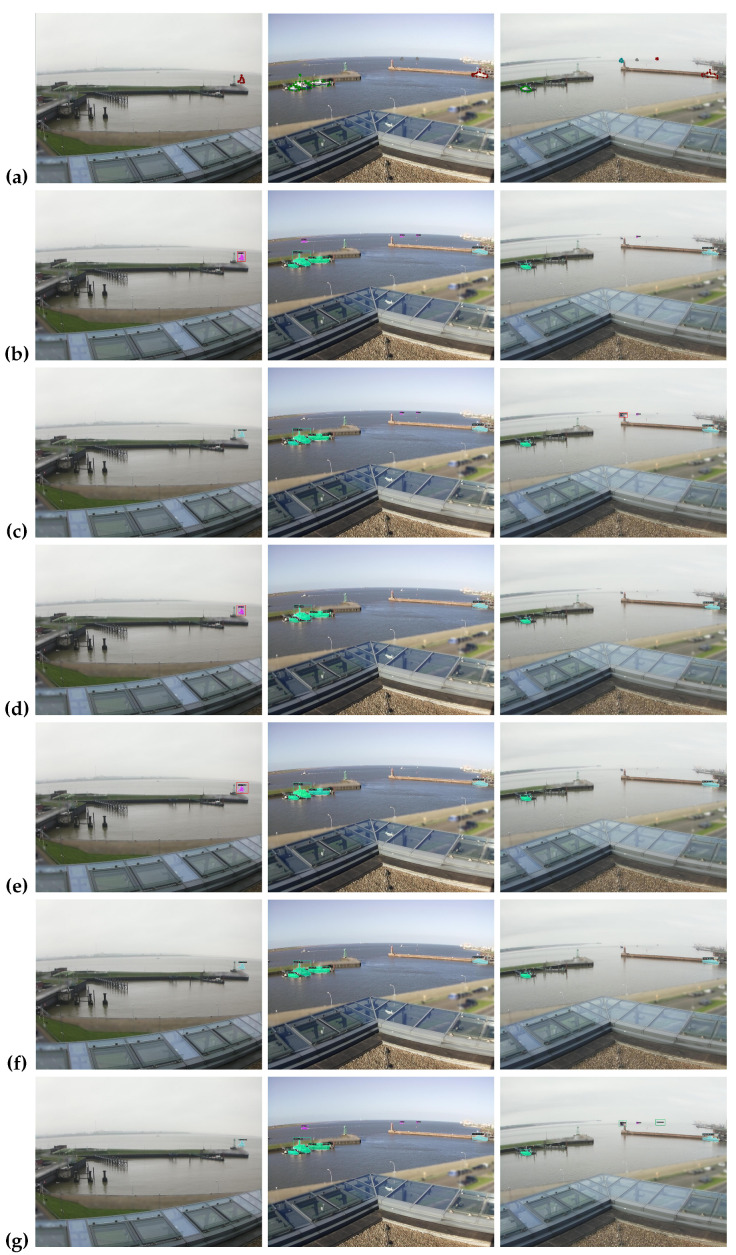
Visual results based on different state-of-the-art methods for the ShipSG dataset. Starting from (**a**), the ground truth, (**b**–**g**) relate to Mask RCNN [1], Cascade Mask RCNN [24], YOLACT [52], SOLO [19], SOLOv2 [53] and ours, respectively. The green box represents correct the class segmentation for the multi-class dataset, whereas the red box indicates inaccurate class detection (best viewed in color).

**Figure 10:**
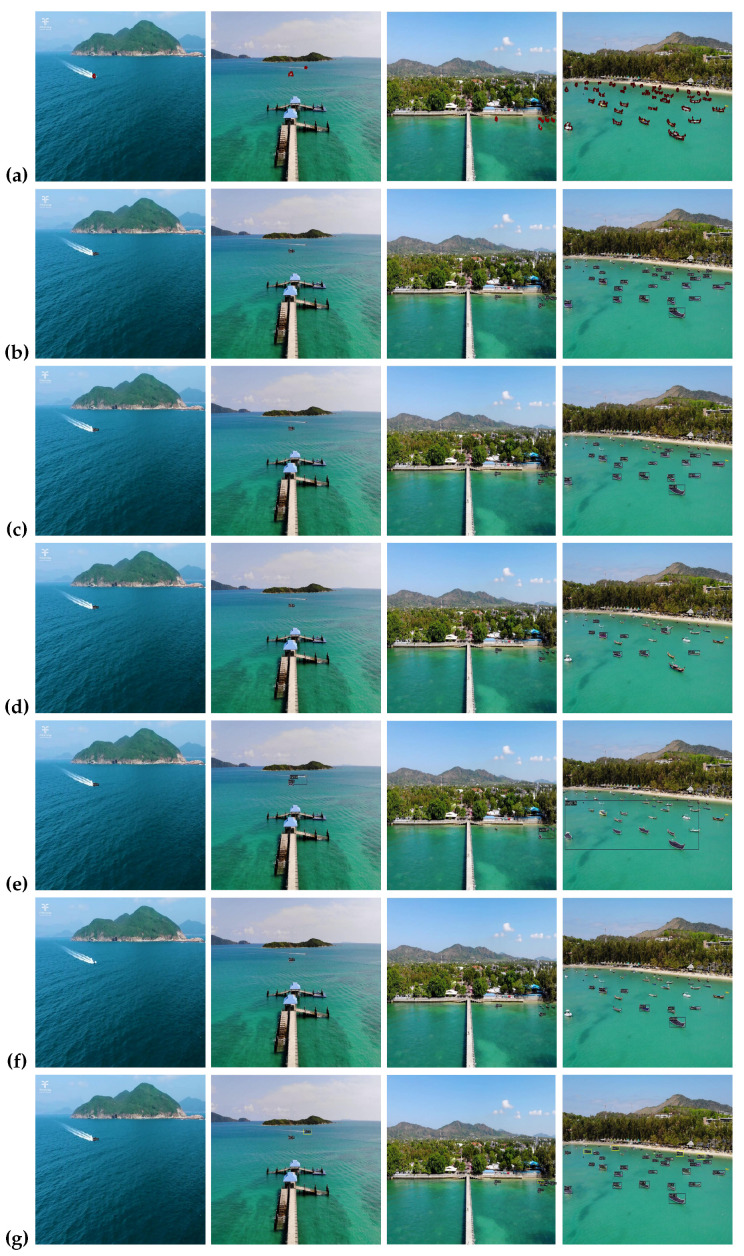
Visual results based on different state-of-the-art methods for the ShipInsSeg dataset. Starting from (**a**), the ground truth, (**b**–**g**) relate to Mask RCNN [1], Cascade Mask RCNN [24], YOLACT [52], SOLO [19], SOLOv2 [53] and ours, respectively. Yellow boxes indicate new ship detection and segmentation (best viewed in color).

**Figure 11:**
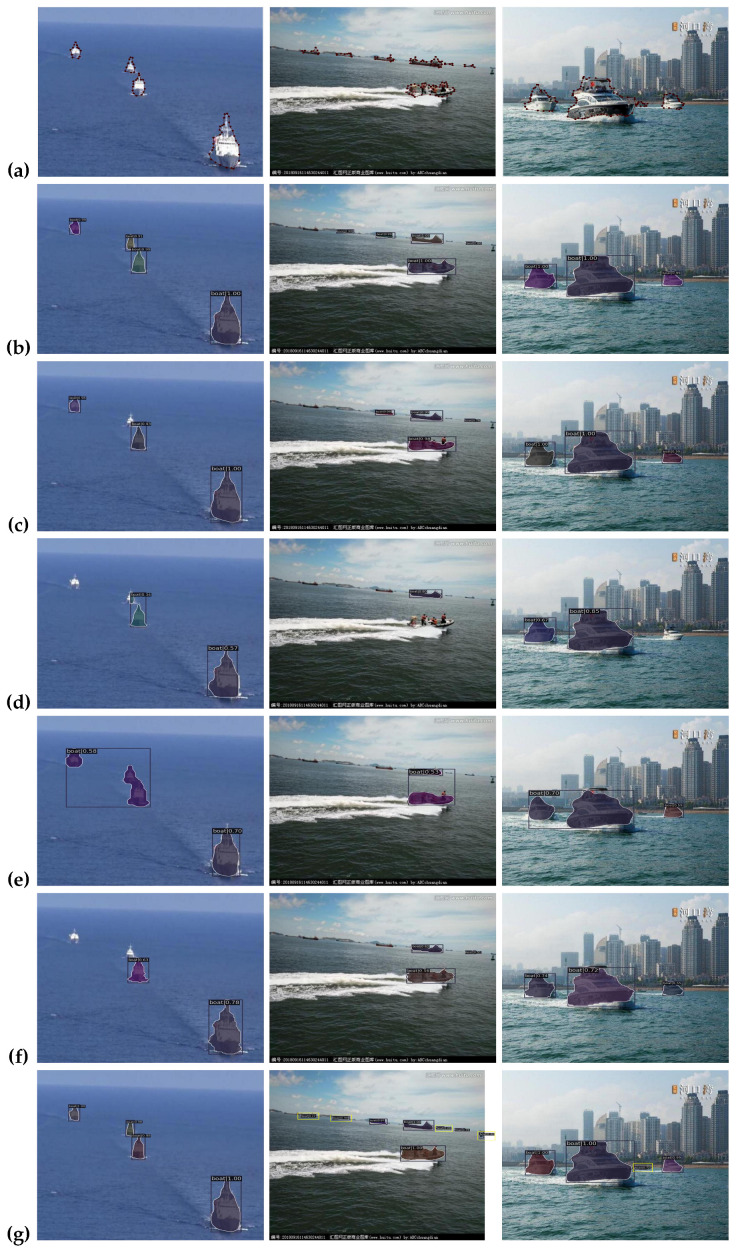
Visual results based on different state-of-the-art methods for the MariBoats dataset. Starting from (**a**), the ground truth, (**b**–**g**) relate to Mask RCNN [1], Cascade Mask RCNN [24], YOLACT [52], SOLO [19], SOLOv2 [53] and ours, respectively. The yellow boxes show new ship detection and segmentation (best viewed in color).

In this section, we discuss the visual comparison of our method on three marine datasets: ShipSG, ShipInsSeg, and MariBoats. We compare five benchmark instance segmentation models arranged accordingly for all datasets, namely, (a) ground truth, (b) Mask RCNN, (c) Cascade Mask RCNN, (d) YOLACT, (e) SOLO, (f) SOLOv2, and (g) ours as shown in Figure 9, Figure 10 and Figure 11, respectively. Based on the visual results obtained from the comprehensive experiments, we demonstrate the effectiveness of our proposed methodology, which is evident in its ability to detect and segment ships.

**ShipSG Dataset**: Figure 9 presents the visual results for performing ship instance segmentation using the ShipSG dataset. We observe that row (c,f,g) performs better when segmenting a single ship by correctly classifying the category, whereas row (b,d,e) fails to identify the correct ship class. In contrast, none of the models demonstrate the capability to segment small ships when segmenting across multiple categories as illustrated in rows (c–f); however, rows (b) and (g) show success in this regard. The performance of our model is substantially enhanced when row (b) is compared in terms of generating precise masks for multiple classes. We also note that, due to the haze, most of the models are unable to identify and segment small ships as illustrated in rows (b–f). However, as demonstrated in row (g), our model is capable of detecting and segmenting small ships into multiple categories.

**ShipInsSeg Dataset**: The visual results of our experiments on the ShigInsSeg dataset are presented in Figure 10. Row (a) illustrates the ground truth images. The visual outcomes from different instance segmentation models are shown in rows (b–g). When compared to other models, our model (g) generates precise edge segmentation and detection results for the same category. Furthermore, given a complex background, we see that our model performs better than the other models and is capable of detecting and segmenting small ships, indicating its applicability and versatility. The newly detected and segmented ships are highlighted in the yellow box.

**MariBoats Dataset**: The visual outcomes of the experiments performed on the MariBoats dataset are depicted in Figure 11. The ground truth images can be seen in row (a). Rows (c), (d), (e), and (f) show that the other cutting-edge instance segmentation models fail to detect and segment ships. Our model (g) achieves superior performance in many circumstances, such as diverse ship sizes, ships located at great distances, and complex backgrounds. Additionally, we highlight our model (g) performance in the yellow box, showing it is capable of detecting and segmenting new ships accurately. In this regard, our model approaches the ground truth more closely.

Finally, the visual results on all three maritime datasets indicate the importance of our proposed technique in effectively identifying and segmenting smaller ships.

## 5. Discussion

The enhanced ASPP feature fusion module improves ship instance segmentation by effectively detecting ships of various sizes, particularly small ones, in complex maritime environments. Utilizing different dilation rates, it captures diverse spatial contexts, allowing the model to focus on fine details and suppress irrelevant background features. Experimental results demonstrate enhanced segmentation accuracy for small ships, reflected in higher mAP scores and visual performance. However, the method increases computational demands, leading to a lower frame per second (FPS) rate that does not meet the real-time performance requirements. This limitation could be addressed in future work by reducing the computational cost and increasing the FPS to achieve real-time capabilities.

## 6. Conclusions

The challenge of maritime surveillance lies in accurately detecting and segmenting small ships, which contains only a few pixels in aerial images due to their distance from the surveillance camera, leads to inaccurate visual information, which makes it difficult for current segmentation techniques to identify small ships against complex backgrounds. In this paper, we propose an enhanced ASPP feature fusion module that is capable of segmenting small ships using different dilation rates and suppressing irrelevant features to focus and locate small ships. We evaluate our method through extensive experiments on the three marine datasets, MariBoats, ShipSG, and ShipInsSeg. Our proposed module outperforms existing methods for segmenting small ships, with considerable increases in precision and recall metrics. Future research on small ship instance segmentation can combine techniques such as generative adversarial networks, transformers, and LLM to enhance the performance of small ships.

## Figures and Tables

**Table 1:** Detailed information on the three marine ship datasets.

Dataset	ShipSG	ShipInsSeg	MariBoats
Small objects	1102	6208	3707
Medium objects	8885	11,313	4693
Large objects	1638	5425	7203
Number of instances	11,625	22,946	15,603
Type	Visible Images	Visible Images	Visible Images
Task	Instance Segmentation	Instance Segmentation	Instance Segmentation

**Table 2:** Instance segmentation performance (mask AP) on ShipSG dataset.

Methods	Backbone	AP	AP_50_	AP_75_	AP_S_	AP_M_	AP_L_	AR	AR_S_	AR_M_	AR_L_	Params (M)	FPS
Mask RCNN [1]	ResNet-50	72.7	97.4	90.6	56.0	73.5	76.3	76.9	62.8	77.9	79.0	43.78	3.7
Mask RCNN [1]	ResNet-101	73.9	97.6	91.7	56.6	74.3	78.2	77.6	64.5	78.1	80.3	62.79	3.3
Cascade Mask RCNN [24]	ResNet-50	73.5	97.7	91.1	55.7	74.1	76.9	76.8	61.3	77.9	78.7	76.85	3.0
Cascade Mask RCNN [24]	ResNet-101	74.3	97.6	92.0	56.5	74.8	78.1	77.6	62.5	78.3	80.4	95.82	2.6
CBAM [30]	ResNet-50	72.2	97.7	90.0	56.5	72.7	74.9	75.9	63.1	76.5	77.6	46.28	2.8
ECA [29]	ResNet-50	72.3	96.8	90.1	55.5	73.1	75.2	76.2	62.7	77.1	77.5	43.79	2.6
SE [28]	ResNet-50	72.2	96.7	90.2	57.4	72.5	75.6	76.2	63.3	76.9	78.1	46.27	2.4
CA [31]	ResNet-50	72.9	97.5	90.4	57.1	73.8	75.4	76.7	63.3	77.7	77.9	43.80	2.1
YOLACT [52]	ResNet-50	54.6	82.9	66.4	21.4	55.3	56.1	64.2	44.6	66.4	67.7	34.77	5.9
YOLACT [52]	ResNet-101	54.4	83.1	66.3	22.5	54.6	57.4	64.3	46.3	66.0	67.3	53.76	4.3
SOLO [19]	ResNet-50	54.9	82.4	64.6	14.5	56.9	62.4	63.1	21.8	65.4	74.0	35.61	3.0
SOLO [19]	ResNet-101	56.1	81.1	65.7	18.2	56.1	64.9	63.9	24.4	65.2	75.7	54.94	2.5
SOLOv2 [53]	ResNet-50	72.7	94.9	87.8	44.7	72.6	82.8	76.7	52.8	76.7	85.4	46.03	5.4
SOLOv2 [53]	ResNet-101	73.3	94.8	88.5	43.2	73.8	**83.7**	77.0	50.9	77.5	86.5	65.66	5.0
**Mask RCNN + Ours**	ResNet-50	73.8	97.5	91.8	60.9	74.8	76.3	77.5	66.4	78.5	78.4	62.75	3.0
**Mask RCNN + Ours**	ResNet-101	75.3	98.3	93.2	61.3	76.2	78.8	78.8	67.2	79.6	81.0	82.75	2.3
**Cascade Mask RCNN + Ours**	ResNet-101	**75.8**	**98.4**	**93.5**	**62.2**	**76.4**	**78.8**	**79.1**	**67.3**	**79.8**	**81.1**	116.3	1.9

**Table 3:** Instance segmentation performance (mask AP) on ShipInsSeg dataset.

Methods	Backbone	AP	AP_50_	AP_75_	AP_S_	AP_M_	AP_L_	AR	AR_S_	AR_M_	AR_L_	Params (M)	FPS
Mask RCNN [1]	ResNet-50	67.7	91.4	76.9	47.1	80.4	87.0	70.4	53.4	82.5	89.9	43.75	11.1
Mask RCNN [1]	ResNet-101	67.7	90.7	77.9	46.9	80.6	87.9	70.4	52.9	82.8	90.6	62.74	9.2
Cascade Mask RCNN [24]	ResNet-50	68.0	92.5	76.9	47.8	80.7	87.1	70.4	53.4	82.6	86.6	76.8	7.6
Cascade Mask RCNN [24]	ResNet-101	68.2	92.5	77.1	47.8	80.8	87.9	70.6	53.5	82.6	90.7	95.79	6.7
CBAM [30]	ResNet-50	65.9	89.0	75.0	44.0	79.0	85.0	69.0	51.4	81.7	89.0	46.24	7.3
ECA [29]	ResNet-50	66.6	90.0	76.0	45.0	80.0	86.0	69.6	51.9	82.3	89.4	43.75	7.1
SE [28]	ResNet-50	66.8	91.0	76.0	46.3	80.0	86.0	69.8	52.3	82.3	89.5	46.24	6.9
CA [31]	ResNet-50	67.0	91.2	76.0	46.7	80.0	86.8	70.0	52.7	82.4	89.8	43.75	7.0
YOLACT [52]	ResNet-50	36.4	62.9	36.1	12.4	54.9	58.5	44.7	20.8	61.5	72.7	34.73	16.7
YOLACT [52]	ResNet-101	41.1	68.9	42.1	13.5	60.4	67.6	48.3	23.9	65.8	75.8	53.72	12.8
SOLO [19]	ResNet-50	29.2	40.3	32.2	3.1	46.2	63.0	33.0	4.4	51.6	70.6	35.89	9.4
SOLO [19]	ResNet-101	28.5	39.5	31.5	2.5	45.2	62.6	32.9	4.1	51.3	71.6	54.89	8.0
SOLOv2 [53]	ResNet-50	48.3	71.7	50.5	16.5	67.8	85.6	52.3	23.1	71.9	88.5	46.0	14.5
SOLOv2 [53]	ResNet-101	49.3	73.4	52.7	17.4	69.6	87.6	53.4	23.8	73.4	90.1	65.59	10.5
**Mask RCNN + Ours**	ResNet-50	67.8	91.5	77.7	47.5	80.7	87.2	70.8	53.6	83.2	90.3	52.75	7.1
**Mask RCNN + Ours**	ResNet-101	67.8	91.8	78.0	47.7	80.3	87.6	70.7	53.2	83.2	90.6	71.75	6.0
**Cascade Mask RCNN + Ours**	ResNet-101	**69.5**	**92.9**	**80.0**	**50.5**	**81.6**	87.9	**72.2**	**56.1**	**83.6**	**90.8**	104.8	4.3

**Table 4:** Instance segmentation performance (mask AP) on MariBoats dataset.

Methods	Backbone	AP	AP_50_	AP_75_	AP_S_	AP_M_	AP_L_	AR	AR_S_	AR_M_	AR_L_	Params (M)	FPS
Mask RCNN [1]	ResNet-50	52.0	80.6	59.5	9.5	31.2	67.8	57.7	20.8	42.0	72.7	43.75	19.50
Mask RCNN [1]	ResNet-101	53.2	80.9	60.1	9.8	31.3	69.7	58.0	19.4	41.2	73.9	62.74	16.0
Cascade Mask RCNN [24]	ResNet-50	52.8	80.3	58.9	9.8	30.5	68.9	58.0	21.4	41.7	73.0	76.8	11.7
Cascade Mask RCNN [24]	ResNet-101	53.5	81.0	60.4	9.9	31.5	70.0	58.3	20.3	41.2	74.0	95.79	10.6
CBAM [30]	ResNet-50	50.9	80.3	57.0	8.4	28.5	67.0	56.9	20.8	40.3	72.0	46.24	12.9
ECA [29]	ResNet-50	51.4	81.2	57.8	10.2	29.4	67.2	57.1	20.8	41.1	72.1	43.75	12.1
SE [28]	ResNet-50	51.2	79.8	57.7	8.7	28.5	67.5	57.1	19.6	40.7	72.4	46.24	11.9
CA [31]	ResNet-50	51.6	80.4	57.9	9.1	28.4	68.0	57.4	20.3	40.6	72.8	43.75	13.0
YOLACT [52]	ResNet-50	36.5	66.4	35.7	2.8	15.0	51.5	46.0	11.0	27.0	61.6	34.73	22.2
YOLACT [52]	ResNet-101	38.5	67.4	38.3	3.0	14.9	54.5	47.3	10.4	27.6	63.8	53.72	18.2
SOLO [19]	ResNet-50	40.6	61.5	46.0	1.2	7.8	61.1	45.4	2.3	15.8	66.9	35.89	17.0
SOLO [19]	ResNet-101	40.8	61.5	46.4	0.8	8.0	61.6	46.0	2.0	15.7	68.0	54.89	14.2
SOLOv2 [53]	ResNet-50	45.9	71.7	49.6	3.2	17.1	65.5	54.1	8.0	31.6	73.9	46.0	21.7
SOLOv2 [53]	ResNet-101	48.3	72.8	51.7	4.0	18.7	68.3	55.7	9.4	31.6	76.0	65.59	19.5
**Mask RCNN + Ours**	ResNet-50	52.4	80.7	59.5	10.5	30.4	68.3	57.7	21.3	41.4	72.8	52.75	10.4
**Mask RCNN + Ours**	ResNet-101	53.5	81.3	60.3	10.2	31.0	69.9	58.3	19.3	41.5	74.2	71.75	8.5
**Cascade Mask RCNN + Ours**	ResNet-101	**54.5**	**81.9**	**62.0**	**10.4**	**32.9**	**70.8**	**59.4**	**22.0**	**43.1**	**74.7**	104.8	6.99

**Table 5:** Comparison between the baseline model Cascade Mask RCNN and our proposed model for correct predictions and other errors on three marine datasets.

Datasets	Method	Correct (%)	Loc (%)	Sim (%)	Other (%)	BG (%)	FN (%)
ShipSG	Cascade Mask RCNN	85.4	0.7	0.2	0	0.1	13.6
	Ours	**86.1**	**0.1**	0.2	0	0.1	**13.5**
ShipInsSeg	Cascade Mask RCNN	92.5	1.1	0	0	1.4	5
	Ours	**92.9**	**1**	0	0	**1.1**	5
MariboatS	Cascade Mask RCNN	81	9.1	0	0	4.9	5
	Ours	**81.9**	**8.2**	0	0	**4**	5.9

**Table 6:** Object detection performance (Box AP) on ShipSG dataset.

Methods	Backbone	AP	AP_50_	AP_75_	AP_S_	AP_M_	AP_L_	AR	AR_S_	AR_M_	AR_L_
Mask RCNN [1]	ResNet-50	79.9	97.1	91.1	61.3	81.3	80.9	84.3	65.2	85.6	87.3
Mask RCNN [1]	ResNet-101	81.3	97.5	91.8	63.0	81.5	83.5	85.4	67.8	85.4	89.8
Cascade Mask RCNN [24]	ResNet-50	83.5	97.4	92.5	64.3	84.4	84.3	86.7	67.7	87.6	90.4
Cascade Mask RCNN [24]	ResNet-101	83.9	97.9	93.3	65.5	85.0	84.9	87.2	68.9	87.7	90.9
CBAM [30]	ResNet-50	79.3	97.5	89.2	62.3	80.6	78.6	83.2	65.9	84.0	85.9
ECA [29]	ResNet-50	79.3	96.8	89.4	61.6	80.7	79.8	83.5	65.4	84.3	87.0
SE [28]	ResNet-50	79.3	96.7	90.3	63.0	80.6	79.8	83.5	65.9	84.1	87.4
CA [31]	ResNet-50	80.0	97.5	90.9	63.8	82.0	79.9	84.2	66.9	85.3	87.3
YOLACT [52]	ResNet-50	48.2	82.9	66.5	21.4	55.3	56.1	58.7	48.8	59.4	57.1
YOLACT [52]	ResNet-101	49.9	82.2	55	35.3	50.9	43.7	61.0	52.2	61.3	59.1
SOLO [19]	ResNet-50	-	-	-	-	-	-	-	-	-	-
SOLO [19]	ResNet-101	-	-	-	-	-	-	-	-	-	-
SOLOv2 [53]	ResNet-50	-	-	-	-	-	-	-	-	-	-
SOLOv2 [53]	ResNet-101	-	-	-	-	-	-	-	-	-	-
**Mask RCNN + Ours**	ResNet-50	81.0	97.8	91.6	66.5	82.6	80.2	85.0	69.6	86.1	87.1
**Mask RCNN + Ours**	ResNet-101	82.3	97.9	92.6	66.8	83.8	81.2	86.2	70.1	86.9	89.5
**Cascade Mask RCNN + Ours**	ResNet-101	**85.0**	**98.2**	**94.4**	**70.0**	**86.2**	84.9	**88.4**	**72.7**	**89.1**	**91.4**

**Table 7:** Object detection performance (Box AP) on ShipInsSeg dataset.

Methods	Backbone	AP	AP_50_	AP_75_	AP_S_	AP_M_	AP_L_	AR	AR_S_	AR_M_	AR_L_
Mask RCNN [1]	ResNet-50	72.0	91.5	81.2	53.9	84.8	89.1	75.0	58.7	87.0	92.7
Mask RCNN [1]	ResNet-101	72.3	90.8	81.3	53.7	84.9	90.1	75.1	58.3	87.3	93.7
Cascade Mask RCNN [24]	ResNet-50	74.2	92.7	83.3	57.4	85.8	91.1	77.0	61.5	88.0	94.5
Cascade Mask RCNN [24]	ResNet-101	74.3	92.7	83.5	57.4	86.1	**92.2**	77.2	61.6	88.2	95.6
CBAM [30]	ResNet-50	70.0	90.2	78.7	51.7	83.4	87.4	73.3	56.3	85.9	91.7
ECA [29]	ResNet-50	71.0	90.4	79.8	52.5	84.2	88.7	74.2	57.3	86.6	92.5
SE [28]	ResNet-50	71.2	91.3	80.0	53.3	84.2	88.2	74.5	58.1	86.5	92.1
CA [31]	ResNet-50	71.5	91.3	81.0	53.8	84.3	88.6	74.7	58.4	86.6	92.5
YOLACT [52]	ResNet-50	34.2	68.4	30.0	20.0	45.0	46.0	44.1	30.6	53.5	60.3
YOLACT [52]	ResNet-101	37.7	72.8	34.5	20.1	50.9	51.5	46.7	31.5	58.4	61.9
SOLO [19]	ResNet-50	-	-	-	-	-	-	-	-	-	-
SOLO [19]	ResNet-101	-	-	-	-	-	-	-	-	-	-
SOLOv2 [53]	ResNet-50	-	-	-	-	-	-	-	-	-	-
SOLOv2 [53]	ResNet-101	-	-	-	-	-	-	-	-	-	-
**Mask RCNN + Ours**	ResNet-50	72.2	91.6	82.1	55.5	84.2	88.7	75.5	59.8	87.0	92.7
**Mask RCNN + Ours**	ResNet-101	72.4	91.0	81.4	54.2	84.5	89.3	75.3	58.7	87.5	93.4
**Cascade Mask RCNN + Ours**	ResNet-101	**75.3**	**93.8**	**84.5**	**59.0**	**86.6**	**91.9**	**78.2**	**63.1**	**88.9**	95.4

**Table 8:** Object detection performance (Box AP) on MariBoats dataset.

Methods	Backbone	AP	AP_50_	AP_75_	AP_S_	AP_M_	AP_L_	AR	AR_S_	AR_M_	AR_L_
Mask RCNN [1]	ResNet-50	53.7	82.0	59.2	14.4	34.7	68.5	60.4	25.1	46.2	74.5
Mask RCNN [1]	ResNet-101	55.9	82.5	59.5	13.8	35.7	71.7	61.1	23.0	45.2	76.4
Cascade Mask RCNN [24]	ResNet-50	59.0	82.6	62.5	15.0	35.9	75.9	64.8	27.5	48.2	80.3
Cascade Mask RCNN [24]	ResNet-101	60.2	83.1	63.8	14.1	38.5	77.0	65.8	25.4	49.7	81.2
CBAM [30]	ResNet-50	53.1	81.8	57.8	12.7	33.0	68.2	59.8	23.9	46.1	73.8
ECA [29]	ResNet-50	53.4	82.1	58.2	13.5	33.6	68.5	59.9	23.8	45.9	74.1
SE [28]	ResNet-50	53.3	81.0	57.8	11.8	33.5	68.7	60.0	22.9	45.4	74.6
CA [31]	ResNet-50	54.1	82.0	59.0	13.2	34.5	69.4	60.5	23.0	47.0	74.9
YOLACT [52]	ResNet-50	33.4	69.6	27.2	5.5	17.0	45.1	44.1	19.7	31.1	54.9
YOLACT [52]	ResNet-101	33.5	70.6	28.1	6.3	17.1	45.1	43.4	10.4	27.6	63.8
SOLO [19]	ResNet-50	-	-	-	-	-	-	-	-	-	-
SOLO [19]	ResNet-101	-	-	-	-	-	-	-	-	-	-
SOLOv2 [53]	ResNet-50	-	-	-	-	-	-	-	-	-	-
SOLOv2 [53]	ResNet-101	-	-	-	-	-	-	-	-	-	-
**Mask RCNN + Ours**	ResNet-50	55.2	82.4	61.1	16.0	34.8	70.3	57.7	25.3	46.4	75.4
**Mask RCNN + Ours**	ResNet-101	57.0	82.8	62.8	14.1	36.2	73.1	62.2	23.1	46.8	77.7
**Cascade Mask RCNN + Ours**	ResNet-101	**60.6**	**83.9**	**65.3**	**15.8**	**39.3**	**77.1**	**66.3**	**27.6**	**50.8**	**81.7**

**Table 9:** Effectiveness of our proposed module on the ShipSG dataset (mask AP).

Methods	AP	AP_50_	AP_75_	AP_S_	AP_M_	AP_L_
ResNet-101	74.30	97.60	92.0	56.5	74.8	78.1
ResNet-101 + Ours at Stage-1	74.38	97.75	92.14	57.89	74.95	78.33
ResNet-101 + Ours at Stage-1 & 2	74.92	97.99	92.59	58.53	75.26	78.57
ResNet-101 + Ours at Stage-1,2 & 3	75.24	98.25	93.10	60.28	75.95	78.69
ResNet-101 + Ours at All-Stage	**75.80**	**98.40**	**93.50**	**62.20**	**76.40**	**78.80**

**Table 10:** Effectiveness of our proposed module on the ShipInsSeg dataset (mask AP).

Methods	AP	AP_50_	AP_75_	AP_S_	AP_M_	AP_L_
ResNet-101	68.2	92.5	77.1	47.8	80.8	87.9
ResNet-101 + Ours at Stage-1	68.34	92.59	77.35	47.99	80.91	87.91
ResNet-101 + Ours at Stage-1 & 2	68.69	92.70	77.80	48.30	80.99	87.94
ResNet-101 + Ours at Stage-1,2 & 3	69.05	92.79	79.49	49.68	81.16	87.92
ResNet-101 + Ours at All-Stage	**69.50**	**92.9**	**80.0**	**50.5**	**81.6**	87.4

**Table 11:** Effectiveness of our proposed module on the MariBoats dataset (mask AP).

Methods	AP	AP_50_	AP_75_	AP_S_	AP_M_	AP_L_
ResNet-101	53.5	81.0	60.4	9.9	31.5	70.0
ResNet-101 + Ours at Stage-1	53.59	81.15	60.55	9.95	31.59	70.05
ResNet-101 + Ours at Stage-1 & 2	53.65	81.31	60.93	9.99	31.73	70.15
ResNet-101 + Ours at Stage-1,2 & 3	53.89	81.56	61.28	10.21	32.51	70.69
ResNet-101 + Ours at All-Stage	**54.5**	**81.9**	**62.0**	**10.4**	**32.9**	**70.8**

## Data Availability

The data included in this manuscript cannot be shared publicly, due to the need to protect the privacy of the included subjects. Data may be shared upon reasonable request to the corresponding author.
